# Multi-CSAR: a multiple reference-based contig scaffolder using algebraic rearrangements

**DOI:** 10.1186/s12918-018-0654-y

**Published:** 2018-12-31

**Authors:** Kun-Tze Chen, Hsin-Ting Shen, Chin Lung Lu

**Affiliations:** Department of Computer Science, National Tsing Hua University, Hsinchu, 30013 Taiwan

**Keywords:** Bioinformatics, Sequencing, Contig, Scaffolding, Multiple reference genomes

## Abstract

**Background:**

One of the important steps in the process of assembling a genome sequence from short reads is scaffolding, in which the contigs in a draft genome are ordered and oriented into scaffolds. Currently, several scaffolding tools based on a single reference genome have been developed. However, a single reference genome may not be sufficient alone for a scaffolder to generate correct scaffolds of a target draft genome, especially when the evolutionary relationship between the target and reference genomes is distant or some rearrangements occur between them. This motivates the need to develop scaffolding tools that can order and orient the contigs of the target genome using multiple reference genomes.

**Results:**

In this work, we utilize a heuristic method to develop a new scaffolder called Multi-CSAR that is able to accurately scaffold a target draft genome based on multiple reference genomes, each of which does not need to be complete. Our experimental results on real datasets show that Multi-CSAR outperforms other two multiple reference-based scaffolding tools, Ragout and MeDuSa, in terms of many average metrics, such as sensitivity, precision, *F*-score, genome coverage, NGA50, scaffold number and running time.

**Conclusions:**

Multi-CSAR is a multiple reference-based scaffolder that can efficiently produce more accurate scaffolds of a target draft genome by referring to multiple complete and/or incomplete genomes of related organisms. Its stand-alone program is available for download at https://github.com/ablab-nthu/Multi-CSAR.

## Background

Although sequencing technologies have greatly advanced in recent years, assembling a genomic sequence from a large number of generated reads still remains a challenging task [1, 2]. Largely because of the presence of repetitive sequences, most of assembled genomes are just *draft* genomes that may be composed of several hundreds of fragmented sequences called *contigs*. The completeness of an assembled genome actually is significant to its downstream analysis and interpretation in many biological applications [3]. For the purpose of producing a more complete genome, the contigs in a draft genome usually are ordered and oriented into larger gap-containing *scaffolds*, in which their gaps can be filled in the subsequent gap-closing process [4].

Although a lot of reference-based scaffolders have been developed, most of them utilize only one genome as the reference to scaffold (i.e., order and orient) the contigs of a target draft genome [5–12]. Actually, the algorithmic methods of all these single reference-based scaffolders can be classified into either alignment-based approaches [5–8] or rearrangement-based approaches [9–12]. For the alignment-based scaffolding approaches, they align contig sequences from a draft genome with the sequence of a reference genome and scaffold these contigs based on their matched positions on the reference genome. As for the rearrangement-based scaffolding approaches, they utilize the information of genome structures to scaffold the contigs in a draft genome such that the order and orientation of conserved genes (or sequence markers) between the scaffolded contigs and the reference genome are as similar as possible. Among the single reference-based scaffolders mentioned above, CAR [11] and CSAR [12] were developed by us based on different rearrangement-based algorithms [13, 14]. In principle, CSAR can be considered as an improved version of CAR, because the reference genome used by CAR is required to be complete, but the one used by CSAR can be incomplete.

In fact, a single reference genome may not be sufficient alone for a scaffolding tool to correctly generate the scaffolds of a target draft genome, especially when the evolutionary relationship between target and reference genomes is distant or some rearrangements (e.g., reversals, transpositions and translocations) occur between them. This motivates the need to develop multiple reference-based scaffolders that can scaffold the contigs of the target draft genome using multiple reference genomes derived from related organisms, which may provide different but complementary types of scaffolding information.

Previously, we utilized a heuristic approach to extend our single reference-based scaffolder CAR to a multiple reference-based scaffolder called Multi-CAR [15] and demonstrated that it performed better than other similar existing tools, such as Ragout [16] and MeDuSa [17], when all the reference genomes are complete. Unlike Ragout and MeDuSa, however, Multi-CAR is not able to accept an incomplete genome as a reference, which ultimately limits its widespread adoption because in practice complete reference genomes are not always available for a target draft genome [18]. In principle, Ragout constructed a breakpoint graph by representing each contig in a target draft genome by two vertices and a contig adjacency supported by reference genomes by an edge with a parsimony cost. The parsimony cost of an edge was computed based on a given phylogenetic tree for the target and reference genomes. Ragout then inferred the contig adjacencies in the target genome from a perfect matching with minimum parsimony cost in the breakpoint graph. By contrast, MeDuSa formulated the contig scaffolding problem as finding a path cover with maximum weight in a scaffolding graph, in which each vertex represents a contig in a target draft genome and each edge represents a contig adjacency with a weight denoting the number of supported reference genomes. Since the computation of an optimal path cover is NP-hard, MeDuSa adopted a 2-approximation algorithm to compute an approximate path cover from the scaffolding graph and then inferred the scaffolds of the target genome from this approximate path cover.

In this study, we further improve our Multi-CAR into a new multiple reference-based scaffolding tool called Multi-CSAR that can utilize multiple complete and/or incomplete genomes as the references to scaffold the contigs of a target draft genome. Our experimental results on real datasets containing multiple incomplete genomes as the references have finally shown that Multi-CSAR still outperforms Ragout and MeDuSa in terms of many average evaluation metrics, such as sensitivity, precision, *F*-score, genome coverage, NGA50, scaffold number and running time.

## Methods

The algorithmic method we use to implement our multiple reference-based scaffolder Multi-CSAR is a graph-based heuristic approach, which (i) utilizes our CSAR [12] to infer single reference-derived scaffolds for a target draft genome based on each of multiple reference genomes, (ii) uses all single reference-derived scaffolds to build an edge-weighted contig adjacency graph, (iii) finds a maximum weighted perfect matching from the contig adjacency graph, and (iv) constructs a multiple reference-derived scaffold of the target draft genome according to the maximum weighted perfect matching. In the following, we describe the details of these four steps in our multiple reference-based scaffolding algorithm.

**Fig. 1:**
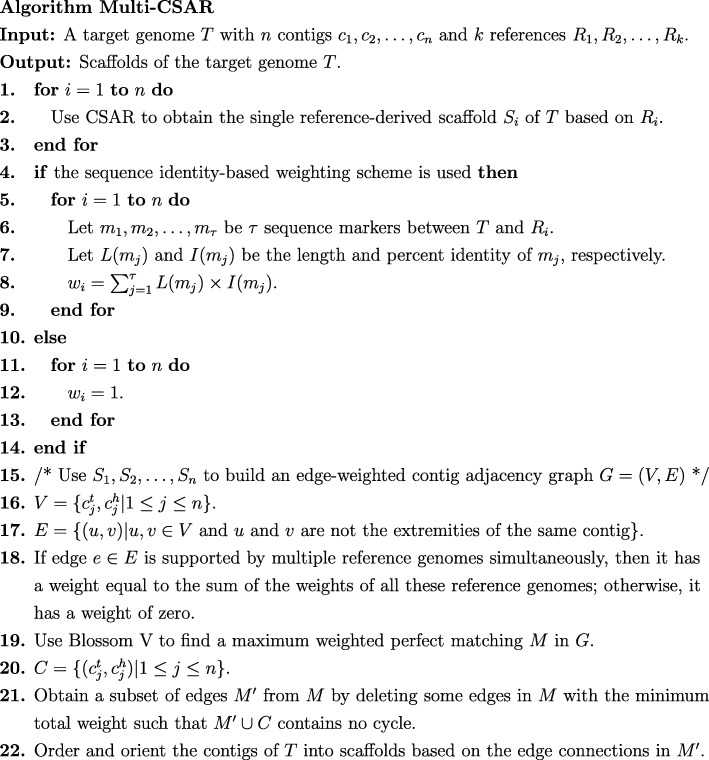
Pseudo-code description for the multiple reference-based scaffolding algorithm we used to implement Multi-CSAR

Suppose that we are given a target draft genome *T* consisting of *n* contigs *c*_1_,*c*_2_,…,*c*_*n*_, as well as *k* references of complete or incomplete genomes *R*_1_,*R*_2_,…,*R*_*k*_ with weights *w*_1_,*w*_2_,…,*w*_*k*_, respectively. We first utilize our single reference-based scaffolder CSAR [12] to obtain a scaffolding result *S*_*i*_ of *T* based on each *R*_*i*_, where 1≤*i*≤*k*. After that, we construct a *contig adjacency graph* *G*=(*V*,*E*) [15], which is an undirected edge-weighted graph as defined below. In principle, a contig *c*_*j*_∈*T*, where 1≤*j*≤*n*, is a fragmented sequence of DNA with two *extremities*, respectively called *head* and *tail*. For our purpose, two vertices, denoted by $c_{j}^{h}$ and $c_{j}^{t}$, are used to represent the head and tail of *c*_*j*_ in *G*, respectively, and an undirected edge is used to connect any two vertices in *G* that are not the extremities from the same contig. In other words, we have $V = \left \{c_{j}^{t},c_{j}^{h} | 1 \le j \le n\right \}$ and *E*={(*u*,*v*)|*u*,*v*∈*V* and both *u* and *v* are not the extremities of the same contig }. We say that an edge in *G* is *supported* by *R*_*i*_ if both of its vertices are adjacent extremities from two different but consecutive contigs in a scaffold of *S*_*i*_. If an edge in *G* can be supported by multiple reference genomes simultaneously, it has a weight equal to the sum of the weights of all these reference genomes. However, if an edge in *G* is not supported by any reference genome, it receives a weight of zero. Next, we use the Blossom V program [19] to find a maximum weighted perfect matching *M* in *G*, where a subset of edges in *G* is called a *perfect matching* if every vertex in *G* is incident to exactly one edge in this subset. Let $C = \left \{\left (c_{j}^{t},c_{j}^{h}\right) | 1 \le j \le n\right \}$ and *M*^′^ be a subset of edges obtained from *M* by deleting some of its edges with the minimum total weight such that *M*^′^∪*C* contains no cycle. Finally, we order and orient the contigs of *T* into scaffolds based on the edge connections in *M*^′^. Note that CSAR was developed by us based on a near-linear time algorithm [14] and the running time of Blossom V is $\mathcal {O}\left (n^{4}\right)$ for a graph with *n* vertices. Therefore, the above multiple reference-based scaffolding method we used to implement Multi-CSAR is a polynomial-time algorithm. We refer the reader to Fig. 1 for its pseudo-code description.

**Fig. 2:**
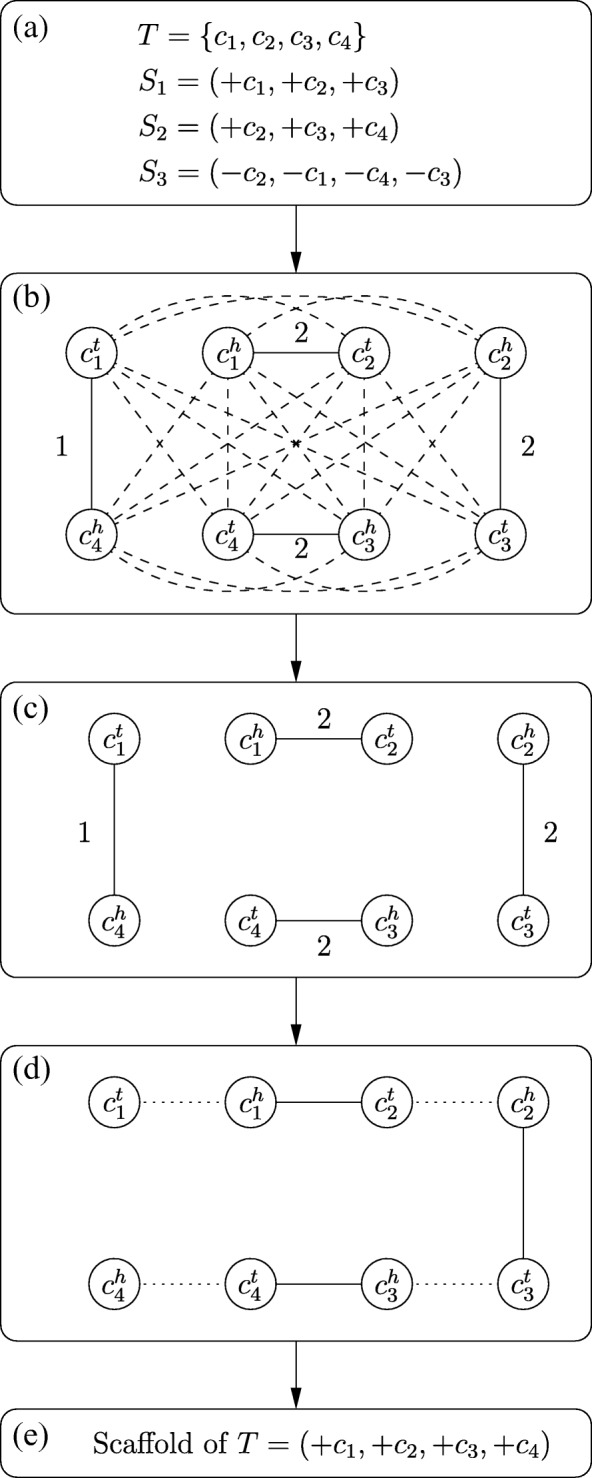
Schematic workflow of Multi-CSAR: **a** A target genome *T*={*c*_1_,*c*_2_,*c*_3_,*c*_4_} and three single reference-derived scaffolds *S*_1_=(+*c*_1_,+*c*_2_,+*c*_3_), *S*_2_=(+*c*_2_,+*c*_3_,+*c*_4_) and *S*_3_=(−*c*_2_,−*c*_1_,−*c*_4_,−*c*_3_) that are assumed to be obtained by applying CSAR on three reference genomes *R*_1_,*R*_2_ and *R*_3_, respectively, with equal weight of one. **b** The contig adjacency graph *G* constructed by using *S*_1_,*S*_2_ and *S*_3_, where the dashed lines denote the edges with zero weight. **c** A maximum weighted perfect matching $M=\left \{\left (c_{1}^{h}, c_{2}^{t}\right), \left (c_{2}^{h}, c_{3}^{t}\right), \left (c_{3}^{h}, c_{4}^{t}\right), \left (c_{4}^{h}, c_{1}^{t}\right)\right \}$ derived by applying Blossom V on *G*. **d** By removing the minimum weighted edge $\left (c_{4}^{h}, c_{1}^{t}\right)$ from *M*, we obtain $M^{\prime }=\{(c_{1}^{h}, c_{2}^{t}), (c_{2}^{h}, c_{3}^{t}), (c_{3}^{h}, c_{4}^{t})\}$ such that *M*^′^∪*C* contains no cycles, where the dotted lines denote the edges in *C*. **e** The final scaffold (+*c*_1_,+*c*_2_,+*c*_3_,+*c*_4_) of *T* constructed based on the edge connections in *M*^′^

Below, we give an example to illustrate how our scaffolding algorithm works (see Fig. 2 for an example). As mentioned previously, a contig is a fragmented sequence of DNA with two extremities, a head and a tail. Given a scaffold, we scan its ordered and oriented contigs in the left-to-right direction. If the tail of a contig, say *c*_*i*_, precedes its head, we write this contig as +*c*_*i*_ in the scaffold; otherwise, we write it as −*c*_*i*_. Suppose that we have the following three scaffolding results *S*_1_=(+*c*_1_,+*c*_2_,+*c*_3_), *S*_2_=(+*c*_2_,+*c*_3_,+*c*_4_) and *S*_3_=(−*c*_2_,−*c*_1_,−*c*_4_,−*c*_3_) that are respectively obtained by applying the CSAR program on a target genome consisting of four contigs *T*={*c*_1_,*c*_2_,*c*_3_,*c*_4_} and three reference genomes *R*_1_,*R*_2_ and *R*_3_ with equal weight of one. We then utilize *S*_1_,*S*_2_ and *S*_3_ to construct the contig adjacency graph *G*=(*V*,*E*) of *T* and apply the Blossom V program on *G* to derive a maximum weighted perfect matching $M=\left \{\left (c_{1}^{h}, c_{2}^{t}\right), \left (c_{2}^{h}, c_{3}^{t}\right), \left (c_{3}^{h}, c_{4}^{t}\right), \left (c_{4}^{h}, c_{1}^{t}\right)\right \}$. By definition, we have $C=\left \{\left (c_{1}^{t}, c_{1}^{h}\right), \left (c_{2}^{t}, c_{2}^{h}\right), \left (c_{3}^{t}, c_{3}^{h}\right), \left (c_{4}^{t}, c_{4}^{h}\right)\right \}$ in this instance. Clearly, *M*∪*C* forms a cycle. In this case, we can remove the minimum weighted edge $\left (c_{4}^{h}, c_{1}^{t}\right)$ from *M* to obtain $M^{\prime }=\left \{\left (c_{1}^{h}, c_{2}^{t}\right), \left (c_{2}^{h}, c_{3}^{t}\right), \left (c_{3}^{h}, c_{4}^{t}\right)\right \}$ such that *M*^′^∪*C* contains no cycles. Finally, we can derive the scaffold (+*c*_1_,+*c*_2_,+*c*_3_,+*c*_4_) of *T*, which is equivalent to (−*c*_4_,−*c*_3_,−*c*_2_,−*c*_1_), according to the edge connections in *M*^′^.

It is worth mentioning that the weights of the reference genomes mentioned before can be derived by Multi-CSAR automatically using the following *sequence identity-based weighting scheme*. As mentioned in our previous study [12], CSAR utilizes either NUCmer or PROmer to identify aligned sequence markers between the target genome *T* and each reference genome *R*_*i*_, where 1≤*i*≤*k*. NUCmer and PROmer are from the MUMmer sequence alignment package [20] that is a set of programs to detect similar regions (i.e. sequence markers) between biological sequences. Particularly, NUCmer detects markers directly on input DNA sequences, while PROmer detects markers on the six-frame protein translation of the input DNA sequences. Suppose that there are *τ* such sequence markers, say *m*_1_,*m*_2_,…,*m*_*τ*_, between *T* and *R*_*i*_. In principle, each such marker *m*_*j*_ actually is a local alignment between *T* and *R*_*i*_, where 1≤*j*≤*τ*. Let *L*(*m*_*j*_) and *I*(*m*_*j*_) be the alignment length and percent identity of *m*_*j*_, respectively. The *weight* of *R*_*i*_ is then given as $w_{i} = \sum _{j=1}^{\tau } L(m_{j}) \times I(m_{j})$. Note that the weights of the reference genomes are all defaulted to one when running Multi-CSAR, unless the sequence identity-based weighting scheme is used.

From algorithmic point of view, Multi-CSAR has the following two new features when compared with its previous version Multi-CAR. First, Multi-CSAR utilizes CSAR, rather than CAR as used in Multi-CAR, to obtain the single reference-derived scaffold of the target draft genome. As mentioned in the introduction, the reference genome used by CAR is required to be complete, but the one used by CSAR can be incomplete. Due to this reason, Multi-CSAR therefore can accept incomplete genomes as references. Second, Multi-CSAR can be run with the sequence identity-based weighting scheme to automatically measure the weight of each reference genome. Generally, the more similar a reference genome is to the target genome, the more weight it receives to support an edge in the contig adjacency graph. In Multi-CAR, however, the weights of all reference genomes must be assigned by the user; otherwise, they are defaulted to one.

## Results

We tested Multi-CSAR, as well as other two multiple reference-based scaffolders Ragout (version 1.0) and MeDuSa (version 1.6), on five real bacterial datasets as shown in Table 1, which were originally prepared and analyzed by Bosi et al. in the study of MeDuSa [17]. Each testing dataset comprises a draft genome to be scaffolded (hereafter called *target genome*) and two or more references of complete and/or incomplete genomes. All the multiple reference-based scaffolders evaluated in this study were run with their default parameters, except Ragout for which a reliable phylogenetic tree for each testing dataset was unknown and hence a star tree was used instead. Consequently, their average performance results over the five bacterial datasets are shown in Table 2. In addition, the average performance results of Multi-CSAR when running with the sequence identity-based weighting scheme are shown in Table 3.

**Table 1:** Summary of the five testing datasets

Organism	No. of replicons	No. of contigs	No. of references	Genome size (Mbp)	GC%
*B. cenocepacia* j2315	4	1,223	4	8.05	65.9
*E. coli* K12	1	451	25	4.64	50.8
*M. tuberculosis*	1	116	13	4.41	65.6
*R. sphaeroides* 2.4.1	7	564	2	4.60	67.4
*S. aureus*	3	170	35	2.90	32.0

**Table 2:** Average performance of the evaluated multiple reference-based scaffolders on the five testing datasets

Scaffolder	Sen.	Pre.	*F*-score	Cov.	NGA50	#Scaf.	Time
Multi-CSAR (NUCmer)	**89.6**	90.8	**90.2**	**93.2**	**1,038,257**	9	**1.7**
Multi-CSAR (PROmer)	89.3	90.4	89.8	92.5	1,016,308	**7**	6.3
Ragout	79.0	**92.5**	84.4	87.4	992,966	84	24.8
MeDuSa	78.2	81.9	80.0	83.3	671,001	26	3.8

The values of sensitivity (abbreviated as ‘Sen.’), precision (abbreviated as ‘Pre.’), *F*-score and genome coverage (abbreviated as ‘Cov.’) are displayed in percentage (%), and the size of NGA50 in base pairs (bp). The column ‘#Scaf.’ gives the number of scaffolds returned by each scaffolder and the column ‘Time’ displays the running time in minutes. The best result in each column is shown in bold

**Table 3:** Average performance of Multi-CSAR on the five testing datasets when using the sequence identity-based weighting scheme

Scaffolder	Sen.	Pre.	*F*-score	Cov.	NGA50	#Scaf.	Time
Multi-CSAR (NUCmer)	**89.9**	**91.3**	**90.6**	**93.5**	**1,046,288**	10	**1.7**
Multi-CSAR (PROmer)	89.4	90.5	89.9	92.8	1,045,489	**7**	6.3

The values of sensitivity (abbreviated as ‘Sen.’), precision (abbreviated as ‘Pre.’), *F*-score and genome coverage (abbreviated as ‘Cov.’) are displayed in percentage (%), and the size of NGA50 in base pairs (bp). The column ‘#Scaf.’ gives the number of resulting scaffolds and the column ‘Time’ displays the running time in minutes. The best result in each column is shown in bold

## Discussion

For the target genome in each testing dataset, Bosi *et al*. also provided a *reference order* of its contigs, which actually was derived from the complete sequence of the target genome and hence can be served as a truth standard in our evaluation. All the tested multiple reference-based scaffolders were evaluated using several different metrics, such as sensitivity, precision, *F*-score, genome coverage, NGA50, scaffold number and running time. In principle, sensitivity, precision and *F*-score are measures to access the accuracy of scaffolds, genome coverage to access the coverage of scaffolds on the target genome, and NGA50 and scaffold number to access the contiguity of scaffolds. In the following, we describe their definitions in detail.

Given two consecutive contigs in a scaffold, they are considered as a *correct* join if they also appear in consecutive order and correct orientation in the reference order. The number of the correct contig joins in a scaffolding result is then called as *true positive* (TP) and the number of the others (i.e., incorrect joins) as *false positive* (FP). Denote by *P* the number of all contig joins in the reference order. The *sensitivity* of a scaffolding result is thus defined as $\frac {\text {TP}}{P}$, its *precision* as $\frac {\text {TP}}{\text {TP}+\text {FP}}$, and its *F*-score (i.e., the harmonic mean of sensitivity and precision) as $\frac {2 \times \text {sensitivity} \times \text {precision}}{\text {sensitivity} + \text {precision}}$ [21]. In principle, *F*-score is a balanced measure between sensitivity and precision and it is high only when both sensitivity and precision are high. To conveniently define the metric of genome coverage below, we assume that the target genome contains only circular DNAs. In this case, therefore, each contig has two neighbor contigs respectively on its both sides. Given a contig in a scaffolding result, if it is correctly joined with its two neighbor contigs on its both sides, its whole length is counted as contributing to the genome coverage (as will be defined later). If this contig is correctly joined with exactly one neighbor contig, half of its length is counted. If it is incorrectly joined with other contigs on its both sides, its length is not counted entirely. The *genome coverage* of a scaffolding result is thus defined as the ratio of the sum of the contig lengths counted using the rules mentioned above to the sum of all contig lengths [10]. Note that if the target genome contains linear DNAs, the first and last contigs located in the reference order of each linear DNA have only one neighbor contig and hence just half of their lengths will be counted in the numerator (if they are correctly joined with their neighbor contigs) and denominator of the genome coverage. The NGA50 value of a scaffolding result is obtained by aligning its scaffolds to the target complete sequence, breaking them at misassembly breakpoints, deleting unaligned regions, and finally computing the NG50 value of the resulting scaffolds that is the size of the smallest scaffold satisfying that 50% of the genome is contained in scaffolds of size NG50 or larger [22].

Clearly, as shown in Table 2, Multi-CSAR running with NUCmer achieves the best scaffolding results in sensitivity, *F*-score, genome coverage, NGA50 and running time, while still exhibiting the second best scaffolding results in precision and scaffold number. On the other hand, when using PROmer to identify sequence markers, Multi-CSAR obtains the best performance in scaffold number, whereas the second best performance in sensitivity, *F*-score, genome coverage and NGA50. From the viewpoint of precision, Ragout performs the best among the evaluated scaffolders. However, its sensitivity is much lower than those obtained by Multi-CSAR running with NUCmer and PROmer, resulting in that its *F*-score is substantially inferior to those of Multi-CSAR with NUCmer and PROmer. In addition, Ragout gives the worst performance in scaffold number and running time. As for MeDuSa, it yields the second best result in running time, but the worst results in sensitivity, precision, *F*-score, genome coverage and NGA50.

On the other hand, it is worth mentioning that, as shown in Table 3, several average accuracy measures of Multi-CSAR, such as sensitivity, precision, *F*-score, genome coverage and NGA50, can be further improved if it is run with the sequence identity-based weighting scheme.

## Conclusions

Scaffolder is a helpful tool for a sequencing project to obtain a more complete sequence of a genome. In this study, we presented Multi-CSAR, an easy-to-use multiple reference-based scaffolder that can efficiently produce more accurate scaffolds of a target draft genome by referring to multiple complete and/or incomplete genomes of related organisms. Multi-CSAR was implemented by a graph-based heuristic approach that utilizes our CSAR to obtain all the single reference-derived scaffolding results, uses them to build an edge-weighted contig adjacency graph, finds a maximum weighted perfect matching from this graph, and finally constructs a multiple reference-derived scaffolding result based on this matching. All the steps in this heuristic approach can be done in polynomial time. Compared with its previous version Multi-CAR, Multi-CSAR has the following two new features: (i) it can accept an incomplete genome as a reference, thus greatly improving its applicability since most of available reference genomes are still incomplete, and (ii) it can automatically derive the supporting weights of reference genomes using a sequence identity-based weighting scheme. By testing on five real prokaryotic datasets containing multiple references of incomplete genomes, our Multi-CSAR indeed outperforms other two multiple reference-based scaffolders Ragout and MeDuSa in terms of average sensitivity, precision, *F*-score, genome coverage, NGA50, scaffold number and running time. In the future, it will be interesting to investigate whether the performance quality of our Multi-CSAR can be further enhanced by incorporating other single reference-based scaffolders, such as OSLay [6], Mauve Aligner [7] and r2cat [8].

## Abbreviations

CAR: Contig assembly using rearrangements
CSAR: Contig scaffolding using algebraic rearrangements
DNA: Deoxyribonucleic acid
FP: False positive
Mbp: Megabase pair
MeDuSa: Multi-draft based scaffolder
Multi-CAR: Multiple reference-based contig assembly using rearrangements
Multi-CSAR: Multiple reference-based contig scaffolder using algebraic rearrangements
MUMmer: Maximal unique match-mer
NG50: Length of the shortest scaffold for which longer and equal length scaffolds cover at least 50% of the genome
NGA50: Analogous to NG50 where the scaffolds are replaced by regions that can be aligned to the target complete sequence
NUCmer: Nucleotide MUMmer
OSLay: Optimal syntenic layouter
PROmer: Protein MUMmer
r2cat: Related reference contig arrangement tool
Ragout: Reference-assisted genome ordering utility
TP: True positive
